# Influence of sedentary behavior on sleep quality in postmenopausal women in high-altitude regions of China: a cross-sectional study

**DOI:** 10.3389/fneur.2024.1476010

**Published:** 2025-01-06

**Authors:** Rilang Leng, Ailin Guo, Guoping Qian, Sujie Mao

**Affiliations:** ^1^Chinese Skating Association, Beijing, China; ^2^Graduate School of Education, University of Exeter, Exeter, United Kingdom; ^3^Faculty of Physical Culture, Gdansk University of Physical Education and Sport, Gdańsk, Poland; ^4^Graduate Development Office, Harbin Sport University, Harbin, China

**Keywords:** sedentary behavior, plateau region, sleep environment, sleep, middle-aged and elderly women

## Abstract

**Objective:**

This study investigates the association between sedentary behavior and sleep quality among postmenopausal women residing in China’s plateau regions. Particular attention is given to moderating effects of age, body mass index (BMI), and sleep environment. This study aims to identify modifiable risk factors influencing sleep quality in this high-altitude population.

**Methods:**

This cross-sectional study focused on postmenopausal women (aged ≥50 years, ≥12 months post-menopause) across four primary plateau regions in China: Qinghai-Tibet, Yunnan-Guizhou, Inner Mongolia, and the Loess Plateau. Sedentary behavior was evaluated with the Older Adults Sedentary Behavior Questionnaire, and sleep quality was assessed with the Pittsburgh Sleep Quality Index. Data analysis encompassed descriptive statistics, correlation analysis, multiple linear regression, and subgroup analyses.

**Results:**

Among the 151 participants (mean age 58.5 years), sedentary behavior was positively correlated with poorer sleep quality (*r* = 0.36, *p* < 0.001). Improvements in the sleep environment were similarly associated with better sleep quality (*r* = 0.29, *p* < 0.001). Multiple linear regression identified sedentary behavior and sleep environment as significant predictors of sleep quality, while other variables showed no significant associations. Subgroup analysis revealed age-specific effects: sedentary behavior had a strong influence on sleep quality in women under 60 years (*r* = 0.36, *p* < 0.01) but demonstrated a weaker, non-significant association in those aged 60 years or older (*p* = 0.062).

**Conclusion:**

Prolonged sedentary behavior is an independent risk factor for reduced sleep quality among postmenopausal women residing in high-altitude regions, while improvements in the sleep environment are positively associated with better sleep quality. The influence of sedentary behavior on sleep quality varies by age groups. These findings highlight the importance of tailored interventions and health policies to improving sleep quality in postmenopausal women living at high altitudes.

## 1 Introduction

Sleep is a fundamental physiological process essential for maintaining health and improving the quality of life (1). High-quality sleep is important in body repair and recovery, particularly in the active cellular repair and regeneration during deep sleep, contributing to the normal functioning across physiological systems (2). Sleep disturbances are prevalent globally, affecting individuals across all age groups (3). A study by the WHO involving 25,916 participants from 14 countries reported that approximately 27% of the population experiences sleep problems (4). Aging is associated with significant changes in sleep structure and quality, making older adults particularly vulnerable to insomnia and shallow sleep (5–7). In China, the prevalence of sleep disorders in the elderly is estimated at 47.2%, and postmenopausal women may face even higher risk, and this risk tends to increase with age (8).

Sleep disorders tend to increase with age (8). Numerous studies have shown that postmenopausal women are particularly susceptible to sleep disturbances, including insomnia, nocturnal awakenings, and difficulty falling asleep, compared to younger women (9–11). The decline in estrogen levels, along with physiological changes such as altered temperature regulation and hormone secretion, contributes to reduced sleep quality (12). Psychological issues such as anxiety and depression are also more prevalent among postmenopausal women and are strongly associated with poor sleep quality (13). Prolonged poor sleep in postmenopausal women can lead to health problems, including cardiovascular issues such as hypertension and coronary heart disease, as well as a weakened immune system, increasing the risk of infections (14). Postmenopausal women living at high altitudes are especially prone to sleep disturbances (15). Environmental factors, such as high altitude, expose postmenopausal women to hypoxia, which leads to adaptive changes in the circulatory system. These changes directly impact blood circulation, negatively affecting brain regulation during sleep (16, 17). Hypoxic conditions may also induce physiological adjustments, including adaptive changes in the circulatory system, influencing the balance of overall blood pressure and blood flow (18). Consequently, postmenopausal women living in high-altitude regions are more likely to experience difficulties falling asleep, nocturnal awakenings, and an overall decline in sleep quality, significantly impacting their quality of life (19, 20).

Existing evidence suggests that sedentary behavior in postmenopausal women at high altitudes is influenced by various factors, including altitude-related challenges, physical health, psychological conditions, and lifestyle habits (15, 21). Evidence indicates that Sedentary behavior is an independent risk factor for sleep disorders (22). Sedentary behavior is defined as any waking behavior characterized by an energy expenditure ≤1.5 METs while in a sitting, reclining, or lying posture. A survey conducted in six low- to middle-income countries reported a significantly higher prevalence of sleep problems in elderly individuals who sit for ≥8 h/day compared to those sitting for ≤4 h/day (23). Prolonged sitting is closely associated with chronic diseases such as cardiovascular diseases, diabetes, and obesity. It negatively affects both physiological functions and mental health (24).

Research has mainly focused on changes in relevant indicators among young populations in high-altitude regions, with limited attention to sleep quality issues in postmenopausal women (25). Evidence on the relationship between sedentary behavior and sleep quality in postmenopausal women at high altitudes is limited, with most existing studies focusing on other populations such as adolescents or specific occupational groups (26–29). To address this gap, this study conducts a cross-sectional investigation to explore the relationship between sedentary behavior and sleep quality in postmenopausal women residing in high-altitude regions. It also aims to examine the impact of sedentary behavior on sleep quality across different characteristics. This study aims to generate evidence that may inform strategies to improve the lifestyle and promote the physical and mental health of postmenopausal women living at high altitudes, ultimately contributing to their quality of life.

This study hypothesizes that prolonged sedentary behavior independently impairs sleep quality in postmenopausal women at high altitudes, with age, BMI, and sleep environment as moderating factors.

## 2 Research methodology

**Figure 1:**
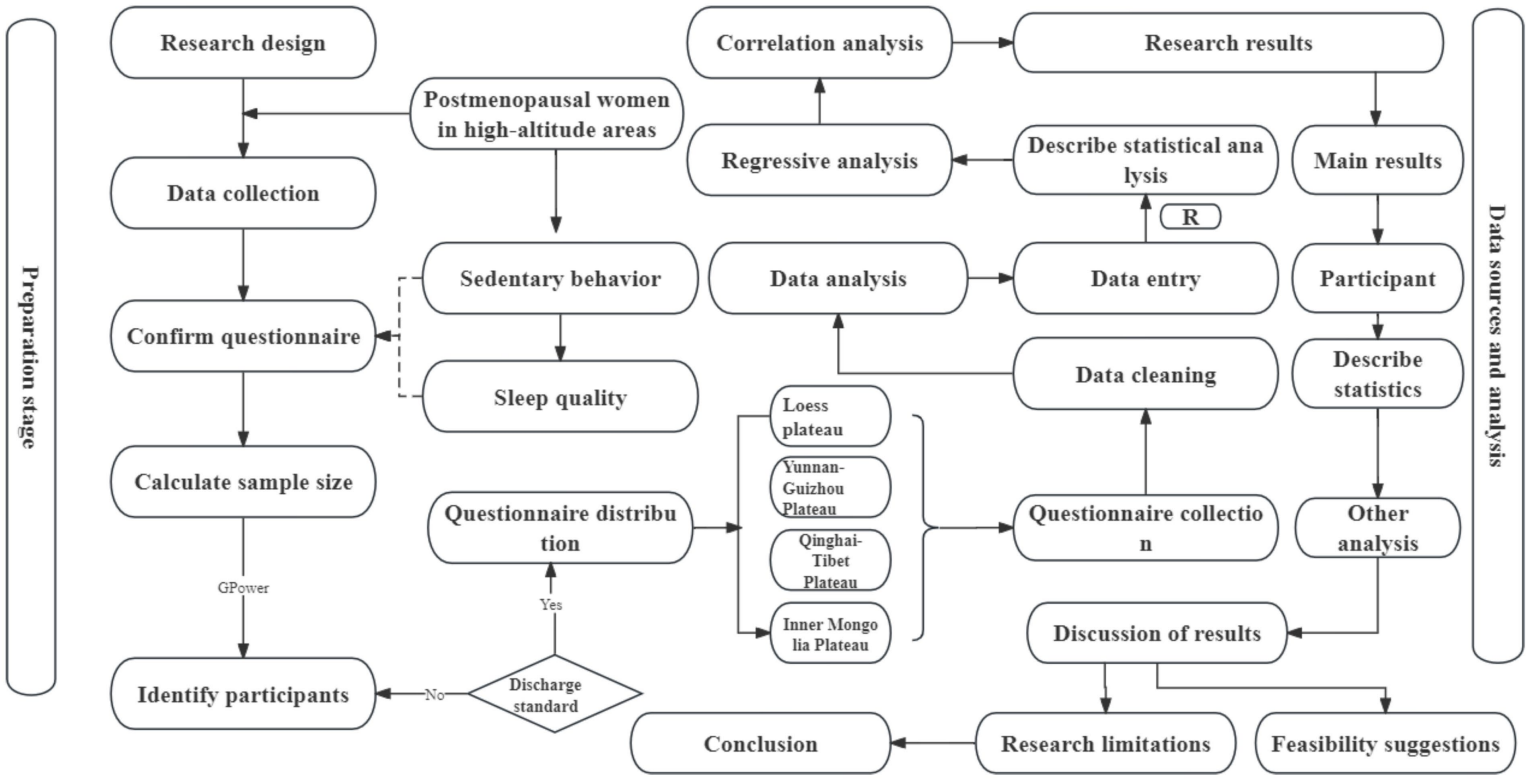
Research design flow chart.

This study investigates the relationship between following the STROBE checklist (30). A cross-sectional design was performed, and data were collected through a questionnaire survey covering the four major high-altitude regions. Statistical analysis was conducted using R software (Figure 1).

### 2.1 Study population and sample selection

#### 2.1.1 Study setting

This study surveyed postmenopausal women in the four major high-altitude regions of China (Qinghai-Tibet, Yunnan-Guizhou, Inner Mongolia, and Loess Plateau), focusing on the relationship between their sedentary behavior and sleep quality. Diverse recruitment methods, including community outreach and online dissemination were employed to include the inclusion of participants from different socioeconomic backgrounds and lifestyles, ensuring the diversity and representativeness of the study. During recruitment, strict inclusion and exclusion criteria were followed to ensure the consistency of the study subjects. Participants completed comprehensive questionnaire surveys covering aspects of sedentary behavior and multiple sleep-related issues. To ensure data integrity and accuracy, necessary follow-up surveys were conducted. The questionnaire distribution period was from March 1, 2023, to April 1, 2023, with the collection period from April 2, 2023, to April 10, 2023.

#### 2.1.2 Inclusion criteria

1. Age ≥ 50 years.
2. At least 12 consecutive months without menstruation, confirming menopause.
3. Resided in high-altitude regions of China for ≥1 year to ensure adaptation.

#### 2.1.3 Exclusion criteria

1. Diagnosed sleep disorders (insomnia, sleep apnea).
2. Severe mental health issues (depression, anxiety).
3. Major chronic diseases (heart disease, cancer) influencing results.
4. Mobility limitations (disability, physical injury).
5. Undergoing treatments affecting sedentary behavior.
6. Significant life changes in the past year impacting sleep or sedentary behavior.

### 2.2 Sample extraction method

This study employed a simple random sampling method to ensure the randomness and representativeness of the sample. The target population was included in the sampling frame, and samples were randomly selected using a computer-generated random number generator. During the sampling process, each postmenopausal woman meeting the inclusion criteria had an equal probability of being selected. This randomsampling method helps minimize selection bias, ensuring that the sample adequately represents the diversity of the target population.

### 2.3 Sample size

We used G-Power software for sample size calculation (31), with parameters set to a one-tailed test, test family as *t-*tests, effect size as 0.3, *α* err prob. as 0.05, and power as 0.95. According to the calculation results, the required sample size for this study is 111 individuals. To enhance the reliability and robustness of the study, 200 questionnaires were distributed to account for potential sample loss, non-response, or incomplete data. This approach ensured that the final sample size meets the research objectives, improving the statistical power and enhancing the reliability and generalizability of the results.

### 2.4 Data statistics

Data were first input and cleaned to exclude incomplete or abnormal records. Descriptive statistics, including mean, standard deviation, and percentages, were employed to thoroughly describe the basic data. To address potential data missing, appropriate imputation and interpolation techniques were used, and data integrity checks were conducted. Correlation analysis was performed using R software to deeply investigate the association between sedentary behavior and sleep quality in postmenopausal women. Regression analysis was employed, incorporating sleep environment, physical activity level, age, and BMI as covariates to assess their potential influence on the relationship between sedentary behavior and sleep quality. The inclusion of these covariates provided a more accurate interpretation of the association between sedentary behavior and sleep quality.

### 2.5 Variable definition and measurement

#### 2.5.1 Main variables

Sedentary behavior: sedentary behavior was assessed using the Older Adults Sedentary Behavior Questionnaire, a customized survey recording daily sedentary time. To meet specific needs, the questionnaire was translated into Chinese, and relevant questions were selected. Previous provided scoring based on the questionnaire results, where higher scores indicate a higher risk level of sedentary behavior (32). The total score (maximum 24 points) was divided into four levels:

Low risk (1–7 points): indicates less sedentary time in daily life with a relatively active lifestyle.

Moderate risk (8–14 points): indicates some sedentary behavior but not reaching a high-risk level.

High risk (15–19 points): indicates frequent sedentary behavior, suggesting a need to increase physical activity.

Very high risk (20–24 points): indicates extremely frequent sedentary behavior, likely to have a negative impact on health, requiring intervention.

Sleep quality: the Pittsburgh sleep quality index (PSQI) was used to measure sleep quality in this study (60). The questionnaire was translated into Chinese, and for authenticity, it was simplified. The scoring system (maximum 21 points) is divided into four levels:

0–4 points (Excellent): represents very good sleep quality.

5–10 points (Good): indicates good sleep quality.

11–15 points (Fair): represents average sleep quality.

16–21 points (Poor): indicates poor sleep quality, requiring improvement.

#### 2.5.2 Covariates

*Sleep environment*: sleep environment quality will be assessed through a sleep environment questionnaire (33), with a total score of 21 points. Lower scores indicate better sleep environment.

*BMI (Body mass index)*: BMI will be calculated based on height and weight measurements obtained during a physical examination.

*Age*: age will be recorded through questionnaire surveys.

*Baseline data*: baseline characteristics will include age, medication usage, place of residence, and cardiovascular treatment status.

#### 2.5.3 Questionnaire design

The translation of the questionnaire in this study adhered to a standardized protocol to ensure scientific rigor and cultural appropriateness. Initial translation was conducted by ALG, a postgraduate with expertise in academic translation. Two domain experts subsequently reviewed the translated text, ensuring the accuracy of terminology and phrasing. A back-translation approach was employed, wherein the Chinese version was retranslated into English and compared with the original to identify and address semantic discrepancies or omissions. Revisions were made based on expert feedback, and the final version was adjusted as needed before its formal implementation in the study.

### 2.6 Quality control

To ensure the accuracy and reliability of the data, strict quality control measures were implemented. All questionnaire distributors were supervised by trained personnel to ensure consistency and standardization in the data collection process. This measure aims to minimize potential human errors and guarantee the scientific validity and credibility of the research results.

### 2.7 Ethical considerations

This study strictly adhered to ethical guidelines and principles outlined in the Helsinki Declaration (34), ensuring that all participants provided informed consent before the study began. The study was approved by the Ethics Association of Nanjing Sport Institute (RT-2023-19) (Appendix 1). The confidentiality of personal information was highly prioritized, and all collected information was used only for academic research purposes. To protect participant privacy, all collected information remained anonymous, and encryption was applied during the data storage phase. Once data analysis was completed, the data were promptly encrypted and, upon completion of the study publication, were destroyed, ensuring maximum protection of participant privacy and personal information security.

## 3 Results

**Figure 2:**
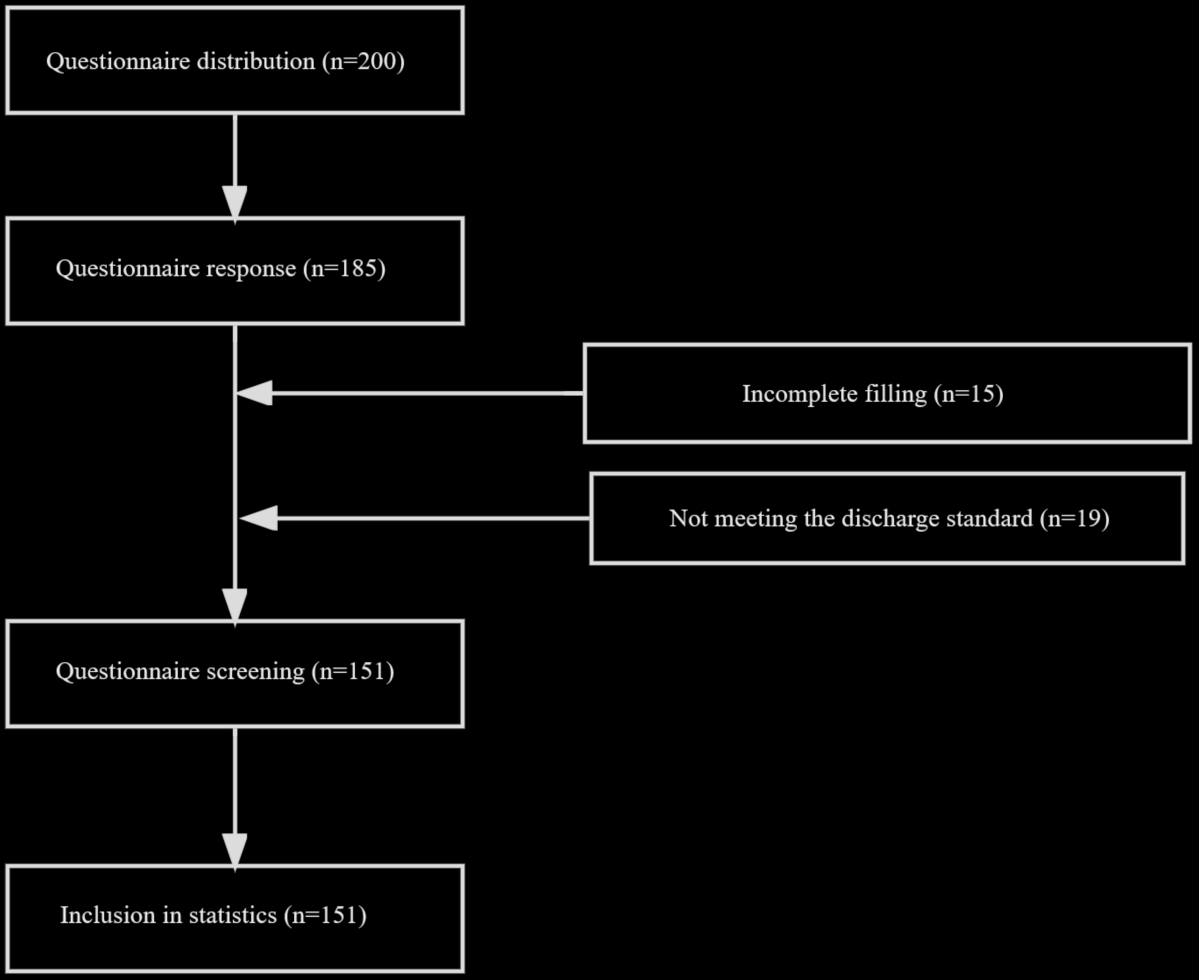
Questionnaire recycling flow chart.

To ensure an adequate sample size, 200 questionnaires were distributed to postmenopausal women residing in the high-altitude regions of China. Successfully, 185 questionnaires were retrieved. However, 15 questionnaires were excluded due to incomplete responses, and 19 were excluded because the participants did not meet the criteria for postmenopausal status. Consequently, 151 complete and usable questionnaires were included in the study (Figure 2 - Questionnaire Retrieval Process).

### 3.1 Sample characteristics

#### 3.1.1 Basic information of participants

The study sample consisted of 151 postmenopausal women from high-altitude regions (Table 1). The average age of participants is 58.51 years, with a maximum age of 63 years. In terms of height, the average is 155.38 cm, with the highest recorded height being 165 cm. The average weight is 64.12 kg, with the highest recorded weight being 80 kg. The average Body Mass Index (BMI) is 26.49, with a standard deviation of 2.60. Among the study population, 53.3% are from the Yunnan-Guizhou Plateau, 18.6% from Inner Mongolia Plateau, 15.3% from the Loess Plateau, and 13.3% from the Qinghai-Tibetan Plateau. Furthermore, 47.7% of participants reported recent use of cardiovascular-related medications, while 52.3% did not use such medications.

**Table 1:** Basic situation description.

Statistic	Mean	SD	Areas	Number
Age	58.47	4.70	Loess Plateau	23
Height	155.39	8.24	Mongolia Plateau	28
Weight	64.08	2.59	Qinghai-Tibet Plateau	20
BMI	26.46	2.59	Yunnan-Guizhou Plateau	80
Number of postmenopausal women				151

#### 3.1.2 Descriptive statistics of sedentary behavior and sleep quality

Based on the results of the translated questionnaire for sedentary behavior (Table 2), 15.89% of the participants were assessed as low-risk, 42.38% as medium-low risk, indicating a relatively moderate level of sedentary behavior. The high-risk group consists of 62 individuals (41.06%), showing a certain degree of risk of sedentary behavior. The very high-risk group comprises only 1 person (0.66%). Sleep quality assessment using the Pittsburgh Sleep Quality Index (PSQI) shows that 6 individuals (3.97%) fall within the 5–10 point range, indicating good sleep quality. A total of 83 individuals (54.97%) scored between 11 and 15 points, categorized as fair sleep quality. A total of 62 individuals (41.06%) scored between 16 and 21 points, indicating poor sleep quality that needs improvement.

**Table 2:** Sedentary and sleep quality Pearson’s correlation description.

Statistic	Value
T	−2.0498
Df	—
*p*	0.04239
Alternative hypothesis	Correlation is not equal to 0
95% CI	−0.3376 to −0.0063
Sample estimate	−0.1769

In summary, most of the postmenopausal women in the high-altitude regions included in this study exhibited moderate to high levels of sedentary behavior, with a relatively small proportion in the high-risk group. Regarding sleep quality, most participants fall into the fair category, with only a small percentage demonstrating good sleep quality.

### 3.2 Correlation analysis

#### 3.2.1 Significance test of variables

Using R software, a significance test was conducted on various variables to evaluate their impact on sleep quality (Table 3). The results showed a significant positive correlation between sedentary behavior and sleep quality (estimated coefficient 0.26, standard error 0.06, *t*-value 4.46, *p =* 0.00002). Age did not show statistical significance, suggesting that, under the condition of other variables remaining constant, its impact on the dependent variable is not significant (estimated coefficient − 0.03, standard error 0.10, *t*-value −0.27, *p =* 0.79). Weight, BMI, and medication use did not reach statistical significance in their impact on the dependent variable. Sleep environment factors, however, showed statistical significance, indicating a significant positive influence on the dependent variable under the condition of other variables remaining constant (estimated coefficient 0.19, standard error 0.06, *t*-value 3.25, *p =* 0.0014).

**Table 3:** One-sample *t*-test power calculation description.

Parameter	Value
Sample size (*n*)	130
Effect size (d)	0.2475732
Significance level	0.05
Desired power	0.8
Alternative hypothesis	Two-sided

These results suggest that sedentary behavior and environmental factors have a statistically significant impact on sleep quality, while other variables did not show significant statistical associations in this sample.

#### 3.2.2 Correlation analysis of sedentary behavior and sleep quality

**Figure 3:**
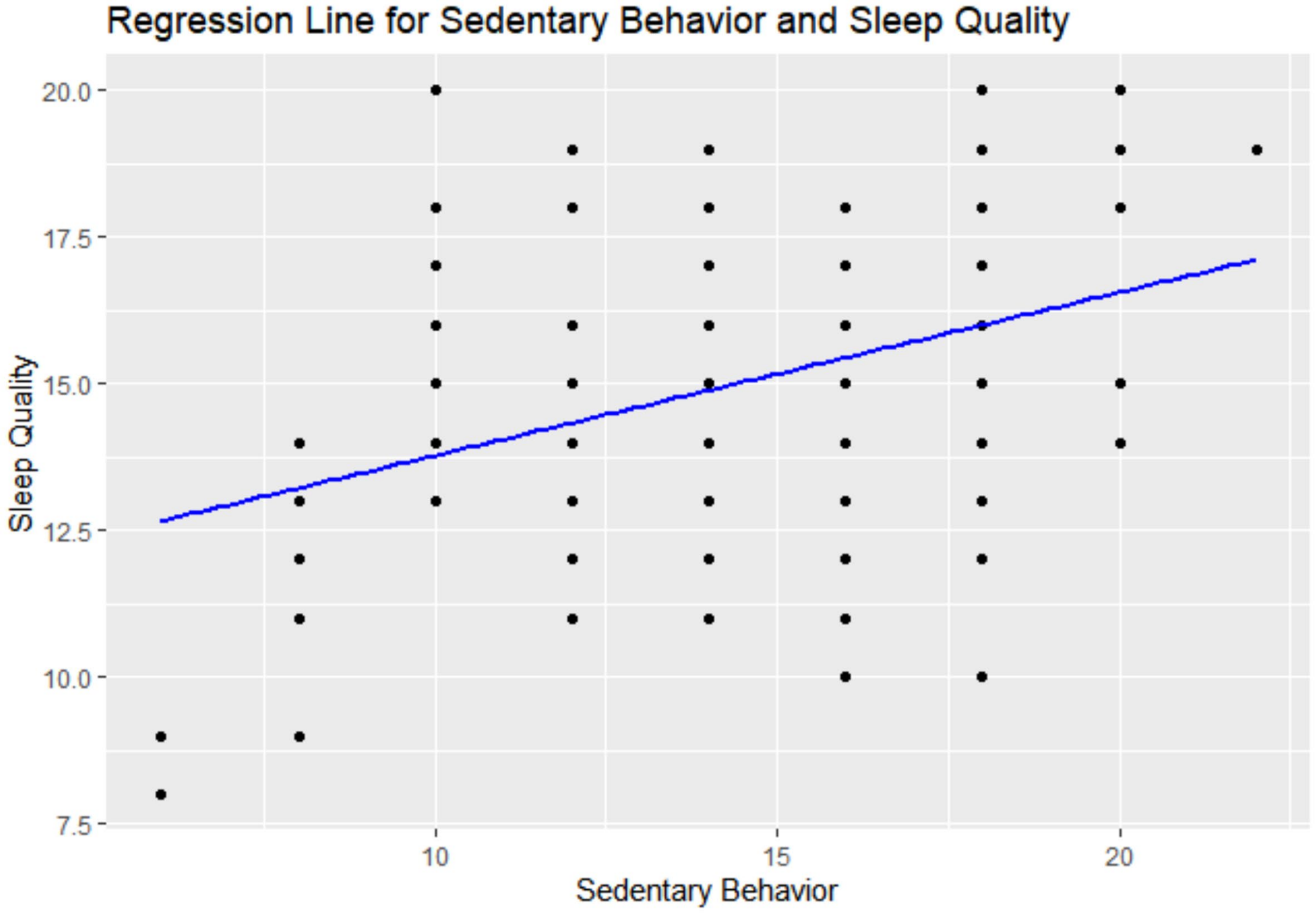
Pseudo-regression line chart.

The correlation analysis revealed a positive correlation between sedentary behavior and sleep quality, with a correlation coefficient of 0.3607 and *p* < 0.001 (statistically significant). As the score for sedentary behavior increases, indicating an increase in sedentary risk, sleep quality scores decrease, reflecting poorer sleep quality. This suggests a significant positive correlation between sedentary behavior and sleep quality, indicating a trend of decreasing sleep quality with prolonged sedentary behavior (Table 4 and Figure 3).

**Table 4:** Linear regression analysis of sedentary behavior and sleep quality.

Statistic	Coefficient	Std. error	*t*-value	Pr(>|t|)
Intercept	7.297760	2.400286	3.040	0.00281 **
Sedentary	0.270567	0.058621	4.616	8.6e-06 ***
age	−0.003461	0.004096	−0.845	0.39948
BMI	0.019912	0.072162	0.276	0.78300
Medicine	0.239053	0.377813	0.633	0.52792
Activity	0.093937	0.078571	1.196	0.23383
Environment	0.186497	0.057296	3.255	0.00141 **
Residual standard error	2.29 on 144 degrees of freedom			
Multiple R-squared	0.2079, Adjusted R-squared: 0.1749			
Adjusted R-squared	0.1749			
*F*-statistic	6.299 on 6 and 144 DF			
*p*-value	6.686e-06			

#### 3.2.3 Correlation analysis of sleep environment and sleep quality

The Correlation analysis between sleep environment and sleep quality showed a correlation coefficient of 0.294973, with *p =* 0.0002362506 (statistically significant). This indicates a significant positive correlation between the two variables, suggesting that lower sleep quality scores are positively associated with better sleep environment scores. Therefore, with improved sleep environment, sleep quality is correspondingly enhanced.

### 3.3 Regression analysis

#### 3.3.1 Multiple linear regression analysis

Using R software, a multiple regression analysis was conducted to explore the complex effects of sedentary behavior, age, body mass index (BMI), medication use, and environmental factors on sleep quality. The estimated coefficient of the intercept term is 7.297760, with a standard error of 2.400286, a *t*-value of 3.040, and *p =* 0.00281, indicating that the intercept term has a statistically significant impact on the dependent variable when other independent variables are held constant (Table 5).

**Table 5:** Regression analysis of sedentary behavior and sleep quality.

Estimate	Std. Error	*t*-value	Pr(>|t|)
Intercept	2.400286	3.040	0.00281 **
Sedentary	0.058621	4.616	8.6e-06 ***
Residual standard error	2.359 on 149 degrees of freedom		
Multiple R-squared	0.1301		
Adjusted R-squared	0.1243		
*F*-statistic	22.28 on 1 and 149 DF		
*p*-value	5.379e-06		

The *p*-values for age, BMI, and medication use are all >0.05, indicating no statistical significance, suggesting that these variables do not significantly affect sleep quality in this study. The estimated coefficient for sedentary behavior is 0.270567, with a standard error of 0.058621, a *t*-value of 4.616, and *p* < 0.001, indicating a significant positive correlation. The estimated coefficient for environmental factors is 0.186497, with a standard error of 0.057296, a *t*-value of 3.255, and *p =* 0.00141, indicating a significant positive correlation between sedentary behavior, environmental factors, and sleep quality. The residual standard error is 2.29, representing the average prediction error of the model for the dependent variable. The multiple R-squared is 0.2079, indicating that the model can explain 20.79% of the variation in sleep quality. The adjusted R-squared is 0.1749, considering the number of independent variables, demonstrating the robustness of the model in explaining the variation in the dependent variable. The F-statistic is 6.299, with a *p*-value of 6.686e-06, indicating that the overall regression model is statistically significant.

#### 3.3.2 Independent impact of sedentary behavior on sleep quality

The regression analysis explored the relationship between sedentary behavior and sleep quality. The estimated coefficient for the regression coefficient is 0.27817, with a standard error of 0.05893, a *t*-value of 4.72, and *p* < 0.001, indicating a positive correlation between sedentary behavior and sleep quality. The multiple R-squared is 0.1301, showing that the regression model can explain 13.01% of the variation in sleep quality. The adjusted R-squared is 0.1243, considering the number of sedentary behaviors, highlighting the robustness of the model in explaining the variation in the dependent variable. The F-statistic is 22.28, with *p* < 0.001, confirming the statistical significance of the overall regression model. The regression analysis results in this study indicate that sedentary behavior statistically has a significant positive impact on sleep quality, and the overall regression model clearly demonstrates this positive relationship. Additionally, we discussed the impact of sedentary behavior and environmental factors on sleep quality (Table 6). The estimated coefficient for the intercept term is 8.59723, with a standard error of 1.08377, a *t*-value of 7.933, and *p* < 0.01, indicating a significant impact of the intercept term on sleep quality when other factors are held constant. The estimated coefficient for sedentary behavior is 0.25402, with a standard error of 0.05736, a *t*-value of 4.429, and *p* < 0.01, indicating an independent and significant positive correlation between sedentary behavior and sleep quality. The overall model explains 19.39% of the variation in the dependent variable. The adjusted R-squared is 0.183, considering the number of independent variables, demonstrating the model’s robustness in explaining the variation in the dependent variable. The F-statistic is 17.79, with *p* < 0.01, indicating that the overall regression model is statistically significant, further emphasizing the significant positive impact of sedentary behavior and environmental factors on sleep quality.

**Table 6:** Coefficients from regression analysis of sedentary and sleep.

Variable	Estimate	Std. Error	*t*-value	Pr(>|t|)
Intercept	8.59723	1.08377	7.933	4.85e-13 ***
Sedentary	0.25402	0.05736	4.429	1.83e-05 ***
Environment	0.19142	0.05595	3.421	0.000806 ***
Signif. codes	0 “***” 0.001 “**” 0.01 “*” 0.05 “.” 0.1 “1”			
Residual standard error	2.279 on 148 degrees of freedom			
Multiple R-squared	0.1939			
Adjusted R-squared	0.183			
*F*-statistic	17.79 on 2 and 148 DF			
*p*-value	1.188e-07			

### 3.4 Subgroup analysis

In this study, through subgroup analysis based on age, BMI, and medication use, we delved into the potential impact of sedentary behavior on sleep quality in postmenopausal women in high-altitude regions of China. This study selected age, BMI, and medication use as subgroup analysis variables based on literature evidence and research design considerations. Age groups were used to explore how different physiological stages affect the relationship between sedentary behavior and sleep quality. BMI groups were chosen due to their potential links with inflammation and metabolic burden. Medication use was included to assess its possible effects on neural regulation and sleep. These variables were selected to provide insights into the mechanisms by which sedentary behavior impacts sleep quality and to support the development of personalized interventions. To comprehensively understand this relationship, participants were divided into two age groups according to China’s definition of the elderly, namely those aged 60 and above and those below 60. Additionally, we used a BMI of 25 as the cutoff to categorize participants into two subgroups: BMI > 25 and BMI ≤ 25, aiming to compare the impact of sedentary behavior on sleep quality under different body mass index conditions. The factor of medication use was also considered, dividing participants into two subgroups: those taking medication and those not taking medication, to explore the potential moderating effect of medication on the relationship between sedentary behavior and sleep quality. Through subgroup analysis, the goal is to provide more specific reference for developing personalized intervention strategies to improve sleep quality in postmenopausal women in high-altitude regions (Table 7).

**Table 7:** Subset analysis by age, BMI, medicine subgroups.

Age	Correlation coefficient	*p*-value
Younger than 60	0.3622	5.16e-05 (Significant)
60 and older	0.3333	0.0623 (not Significant)
BMI subgroup		
BMI		
BMI > 24.9	0.3155	0.000833 (Significant)
BMI ≤ 24.9	0.4798	0.00130 (Significant)
Medicine subgroup		
Medicine		
Medicine = 1(take)	0.3513	0.00150 (Significant)
Medicine = 2(not taking)	0.3739	0.00121 (Significant)

In the subgroup analysis based on age, we explored the relationship between sedentary behavior and sleep quality. In the age group <60, we observed a significant positive correlation between sedentary behavior and sleep quality, with a correlation coefficient of 0.3622 and *p* < 0.01, which is statistically significant. However, in the age group 60 and above, although the correlation coefficient is positive, *p =* 0.0623, it did not reach significance, and the significant association in this age group cannot be confirmed. This suggests that age may influence the relationship between sedentary behavior and sleep quality, warranting further in-depth research to understand this phenomenon.

In the subgroup analysis based on BMI, we investigated the impact of sedentary behavior on sleep quality at different BMI levels. In the BMI > 25 group, we observed a significant positive correlation between sedentary behavior and sleep quality, with a correlation coefficient of 0.3155 and *p =* 0.000833, strongly supporting the existence of this association. In the BMI ≤ 25 group, we similarly observed a significant positive correlation between sedentary behavior and sleep quality, with a correlation coefficient of 0.4798 and *p =* 0.00130. This indicates that regardless of BMI level, sedentary behavior has a significant positive impact on sleep quality.

In the subgroup analysis based on medication use, we studied the impact of medication use on the relationship between sedentary behavior and sleep quality. In the group taking medication, we observed a significant positive correlation between sedentary behavior and sleep quality, with a correlation coefficient of 0.3513 and *p =* 0.00150. In the group not taking medication, we also observed a significant positive correlation between sedentary behavior and sleep quality, with a correlation coefficient of 0.3739 and *p =* 0.00121. This suggests that regardless of medication use, sedentary behavior has a significant positive impact on sleep quality, indicating that medication use may not be a significant influencing factor in this association.

### 3.5 Overall model and variable significance analysis

#### 3.5.1 Overall model significance test

In the significance test of the overall model, we assessed the statistical significance of the regression model as a whole using the F-statistic, with an F-statistic value of 6.101861 (Table 8). This result emphasizes that, considering all independent variables in the study, the regression model has a certain explanatory power for the variation in sleep quality. This further confirms that the regression model in this study fits the observed data well and highlights the significant impact of sedentary behavior on sleep quality.

**Table 8:** Overall model significance test.

Statistics	Value
F-statistic	6.101861
Numdf	6.000000
Dendf	144.000000

#### 3.5.2 Significance test results of variables

In this study, a significance test of multiple independent variables’ regression models was conducted to investigate the significance of each variable (Table 9). The results show that in the regression model for sleep quality in postmenopausal women in high-altitude areas, the intercept term is not significant, indicating a relatively weak influence of the intercept term on sleep quality when considering other variables. Sedentary behavior shows a significant positive correlation, with an estimated coefficient of 0.26, a standard error of 0.06, a *t*-value of 4.46, and *p =* 0.00001663, indicating a significant correlation between increased sedentary behavior and improved sleep quality. Age, weight, BMI, and medication use did not show significant predictive power in the model, as their *p*-values are relatively high. Environmental factors significantly affect sleep quality, with an estimated coefficient of 0.19, a standard error of 0.06, a *t*-value of 3.25, and *p =* 0.0014, indicating a significant positive correlation between environmental factors and sleep quality. The model explains about 3.25% of the variation in sleep quality, and sedentary behavior and environmental factors show significant effects in this model.

**Table 9:** Significance test results for each variable.

Variable	Estimate	Std. error	*t*-value	Pr(>|t|)
Intercept	9.79031976	6.89015103	1.4209151	0.1575033
Sedentary	0.26025029	0.05839872	4.4564381	0.00001663
Age	−0.02613746	0.09638630	−0.2711740	0.7866456
Weight	0.04854856	0.05269377	0.9213339	0.3584171
BMI	−0.12304675	0.16539701	−0.7439478	0.4581203
Medicine	0.28782092	0.37809298	0.7612438	0.4477563
Environment	0.18925187	0.05825343	3.2487675	0.001442608

#### 3.5.3 VIF value test

The Variance Inflation Factor (VIF) was used to assess multicollinearity among independent variables in a regression model. A higher VIF indicates stronger correlation among variables, potentially affecting the interpretability and stability of the model. The results (Table 10) show that, except for weight and BMI, which have relatively high VIF values (>5), the VIF values for other variables are relatively low, indicating small correlations among them and a relatively small impact on the model.

**Table 10:** VIF value.

Variable	Value
Sedentary	1.035586
Age	1.185817
Weight	5.305533
BMI	5.240091
Medicine	1.020182
Environment	1.082842

## 4 Discussion

This study highlights the independent negative impact of prolonged sedentary behavior on sleep quality among postmenopausal women in high-altitude regions. Regression analysis confirmed this association, and environmental factors were identified as significant contributors. Subgroup analysis further showed that the effect of sedentary behavior on sleep quality varied by age, BMI, and medication use.

Subgroup analysis revealed a stronger association between sedentary behavior and sleep quality in younger postmenopausal women (<60 years), likely due to higher metabolic activity and energy balance demands (35). Among women with higher BMI, the negative impact of sedentary behavior on sleep quality was more pronounced, reflecting exacerbated inflammation and circulatory dysfunction in this group (36).

Postmenopausal women are influenced by multiple factors, including hormonal changes, psychological health status, lifestyle, and life event-related stress (37). The decline in estrogen levels disrupts sleep cycles, affecting both sleep initiation and maintenance. Psychological conditions such as anxiety and depression, combined with life stress and adjustments, directly contribute to reduced sleep quality. A meta-analysis of surveys on sedentary behavior in older adults across seven countries revealed that nearly 60% spend more than 4 h sitting, and 67% sit for over 8.5 h a day. Prolonged sedentary behavior has been identified as an independent risk factor for poor sleep quality, along with hypertension, diabetes, and obesity (23). Another study suggested that individuals with longer sedentary times commonly experience sleep disturbances such as insomnia, shorter sleep duration, and lower sleep efficiency (38). Furthermore, research indicates a bidirectional relationship between sedentary behavior and poor sleep quality, leading to a vicious cycle (39, 40). These findings align with the results of this study, providing empirical support for exploring the mechanism by which sedentary behavior affects sleep quality.

A recent cross-sectional study by Zhang et al. (41) on the sleep quality of university freshmen in high-altitude regions found that approximately 14.8% of freshmen experienced sleep disorders, significantly reducing their quality of life (41). This prevalence was higher than that observed in the same age group in the United States, Japan, and Arabia. The study also identified characteristics such as insufficient physical activity and sedentary behavior among students with sleep disorders (41). Another study by Jafarian assessed subjective sleep during ascent to 3,500 m in 100 participants, found a significant decline in sleep quality due to reduced oxygen levels and temperature fluctuations. Studies revealed that reveals a significant association between sedentary behavior and decreased sleep quality in postmenopausal women in high-altitude areas (15, 22). A trial simulating high-altitude sleep quality found that a sedentary lifestyle in low-oxygen conditions reduces total sleep time, sleep efficiency, slow-wave sleep, and rapid eye movement sleep, exacerbating symptoms such as depression, anger, and fatigue (42). Similarly, a randomized controlled trial by Fabries et al. (43) divided participants into two groups, simulating high- and normal- altitude regions, to assess the impact of sedentary behavior and physical activity on sleep quality under hypoxic conditions (43). The study found that sedentary behavior negatively affected sleep quality under hypoxic conditions, while engaging in physical activity improved sleep efficiency. These findings indicated that different lifestyles have a significant impact on sleep quality under hypoxic conditions, and sedentary behavior may exacerbate sleep problems. This finding aligns with the results of this study, emphasizing the adverse effects of sedentary behavior on sleep quality in high-altitude regions facing hypoxic environments (42).

The hypoxic environment at high altitudes may induce adaptive changes in the circulatory system. Prolonged sitting impairs blood circulation, reducing the transport of oxygen and nutrients, including to the brain, thereby interfering with sleep-related regulatory processes (44). In hypoxic conditions, physiological changes such as increased blood viscosity occur (45). Prolonged sitting contributes to blood stagnation, increasing viscosity and hindering blood flow. Hypoxia may also disrupt vascular tone, impairing the ability of blood vessels to adjust their diameter to meet physiological demands. Prolonged sitting exacerbates this imbalance, affecting blood flow (46). Additionally, it may elevate blood pressure, especially in a hypoxic environment, due to increased circulatory burden. Prolonged sitting may also disrupt adaptive cardiovascular changes, affecting hemodynamic balance (39, 40, 47). The combined effects of these mechanisms may impair circulatory adaptability and disrupt sleep regulation in postmenopausal women at high altitudes, weakening compensatory capacity during altitude adaptation and increasing the risk of sleep interruptions and reduced sleep efficiency (48).

With the decline in estrogen levels in postmenopausal women, the hormonal system may experience fluctuations in several hormones (49), including melatonin, playing crucial roles in regulating the biological clock, sleep cycles, and sleep depth. Estrogen maintains the menstrual cycle and protects bone density in the female reproductive system, regulating cholesterol levels (50). After menopause, the decline in estrogen levels may lead to physiological imbalances, affecting sleep cycles and quality. Progesterone plays a critical role in maintaining pregnancy and the uterine lining during the second phase of the menstrual cycle. After menopause, fluctuations in progesterone levels may have potential negative effects on sleep (51). Existing studies have demonstrated a correlation between prolonged sitting and hormonal imbalance, especially in hypoxic high-altitude environments. The body may adaptively adjust hormone production and release to maintain physiological balance, and prolonged sitting may disrupt this balance, leading to abnormal hormone fluctuations. Postmenopausal women are already in a relatively unstable hormonal state, and the dual impact of the hypoxic environment and prolonged sitting in high-altitude areas may exacerbate this instability (52). This may manifest as more pronounced hormone fluctuations, negatively affecting sleep regulation. Considering the combined impact of the hypoxic environment and prolonged sitting on hormonal regulation in postmenopausal women in high-altitude areas provides a more comprehensive understanding of their combined effects on sleep quality (53).

Prolonged sitting in postmenopausal women may lead to metabolic disruptions, including unstable blood sugar levels. In high-altitude areas, due to the hypoxic environment, the body requires more energy to maintain basic physiological processes. However, prolonged sitting may reduce energy expenditure, affecting the body’s metabolic balance (54). Prolonged sitting may decrease physical activity, leading to reduced energy consumption. This may hinder the body’s ability to adapt to high-altitude conditions or result in metabolic disruptions, causing unstable blood sugar levels, leading to nighttime awakenings and sleep interruptions, affecting sleep depth and continuity, thereby negatively impacting sleep quality (55). Additionally, prolonged sitting may cause a dysregulation of the sympathetic and parasympathetic nervous systems, affecting normal nervous system functioning. This imbalance in the nervous system may induce excessive stress responses within the body, negatively impacting the neural regulatory processes of falling asleep and maintaining sleep. A balanced interaction between the sympathetic and parasympathetic nervous systems is crucial for maintaining good sleep (56). Activation of the sympathetic nervous system is associated with stress responses, while the parasympathetic nervous system contributes to bodily relaxation and sleep onset. Prolonged sitting may lead to a lack of coordination between these two neural systems, resulting in a relatively heightened activity of the sympathetic nervous system and a relative insufficiency of the parasympathetic nervous system. This imbalance in the neural systems could potentially place postmenopausal women at a higher risk of experiencing a tense and excited state. It may also reduce the efficiency of relaxation mechanisms, making it difficult for the body to transition into a restful state. Prolonged sitting for extended periods may keep the body in a static position, particularly in the support of the trunk and neck, potentially causing muscle stiffness and discomfort (57). Due to the adaptive changes in the respiratory system caused by hypoxia, the demand for oxygen during respiration may become more sensitive. When the body is in an uncomfortable posture or experiences muscle tension, the respiratory system may need more effort to maintain normal respiration. This may result in postmenopausal women experiencing impaired or irregular breathing during sleep in high-altitude areas, leading to issues such as respiratory pauses, snoring, or other respiratory-related problems, impacting sleep quality.

Reducing sedentary behavior is a practical strategy to improve sleep quality in postmenopausal women in high-altitude regions. Regular light physical activities, such as walking or stretching, can promote blood circulation and oxygen transport (58). Enhancing the sleep environment with indoor oxygen supply devices, improved sound insulation, and temperature regulation may further mitigate hypoxia-related sleep disturbances (59). Public health policies should address the specific needs of this vulnerable population, promoting multi-level interventions to improve their health and quality of life.

This study highlights the importance of addressing sedentary behavior and sleep disturbances in postmenopausal women living in high-altitude regions within a public health framework. Sedentary behavior is a growing global issue linked to metabolic disorders, cardiovascular diseases, and sleep disturbances, which significantly affect quality of life and burden healthcare systems. The unique challenges of high-altitude environments, such as hypoxia, reduced oxygen transport, and increased circulatory strain, amplify these risks and underscore the need for targeted interventions. Cultural and lifestyle differences among high-altitude regions, including dietary habits, daily physical activity, and environmental adaptation, may influence the generalizability of findings. Future longitudinal studies should explore these regional differences and examine the causal relationship between sedentary behavior and sleep disturbances. Intervention trials, particularly those integrating light physical activity and environmental improvements, are crucial for developing effective public health strategies for this vulnerable population.

## 5 Limitations of the study

1. The study focused on postmenopausal women in the four major plateaus of China. However, significant differences in lifestyle, culture, and climate across these regions may potentially impact the consistency of research results.
2. The research adopted a cross-sectional design, providing data only at a specific point in time. This design makes it challenging to capture the long-term dynamic relationship between sedentary behavior and sleep quality. Future studies could consider employing a longitudinal research design to track participants’ changes over time.
3. Self-report bias: the study used a questionnaire survey to collect data, introducing the possibility of self-report bias. Participants’ responses may be influenced by subjective memory and social expectations. Future research could enhance objectivity and accuracy by incorporating objective measurement tools such as accelerometers and sleep monitoring devices.
4. Potential unconsidered confounding variables: despite considering some covariates like sleep environment, physical activity levels, age, and BMI, there may still be other unaccounted confounding variables that could influence the relationship between sedentary behavior and sleep quality.
5. Sample size limitations: although the distribution of 200 questionnaires was determined through sample size calculations, factors such as sample loss, non-response, or incomplete data may result in a smaller effective sample size. This could impact the statistical power of the study and the robustness of conclusions.

## 6 Conclusion

This study investigated the relationship between sedentary behavior and sleep quality in postmenopausal women in high-altitude areas of China, considering the influence of other relevant factors. Correlation analysis indicated a significant positive association between sedentary behavior and sleep quality, suggesting that prolonged sitting may be associated with poorer sleep quality. Regression analysis confirmed that sedentary behavior independently and positively influences sleep quality. Subgroup analysis revealed variations in the impact of sedentary behavior on sleep quality across different age groups. Additionally, the study highlighted the positive influence of the sleep environment on sleep quality, suggesting that improvements in the environment may contribute to enhancing sleep quality. Both sedentary behavior and sleep environment had a significant positive impact on the sleep quality of postmenopausal women in high-altitude areas, providing a basis for developing personalized intervention strategies. Differences in subgroups suggest that factors such as age, BMI, and medication use may play a moderating role in this relationship, warranting further in-depth research to explain these variances. Overall, the study provides a comprehensive understanding of sleep health in postmenopausal women in high-altitude areas, forming a foundation for intervention strategies and health policies.

## Data Availability

The raw data supporting the conclusions of this article will be made available by the authors, without undue reservation.
