# Supplementary material for: Influence of sedentary behavior on sleep quality in postmenopausal women in high-altitude regions of China: a cross-sectional study
Source: Front Neurol. 2025 Jan 6;15:1476010. doi: 10.3389/fneur.2024.1476010 (PMC11743715; doi:10.3389/fneur.2024.1476010)
Supplement: Supplementary file 1 [file Presentation_1.pdf]

南京体育学院人体实验伦理委员会

人体实验研究伦理审查批件

（项目申请用）

编号（ RT-2023-19 ）

项目名称	中国高原地区绝经后妇女久坐行为对睡眠质量的影响，一项横断面研究		
项目类别	A. 人体运动试验 B. 新技术应用 C. 人体标本收集<br>D. 其他（请注明）：√ 问卷调查		
申请项目来源	自筹项目	申请经费	-
系/中心	学科建设办公室		
项目负责人	郭修金	职　称	教授

项目负责人的担保书：

本人保证本人及所有参与本项目研究的实验人员将严格遵循《南京体育学院人体实验伦理委员会章程》，自觉遵守人体实验伦理原则，随时接受南京体育学院人体实验伦理委员会的监督与检查，如违反规定，本人将承担相应的责任并接受处罚。

项目负责人（签名）：[signature] 日期：20230120

审批意见：

南京体育学院人体实验伦理委员会主任委员/副主任委员（签名）：[signature]

（盖章）

南京体育学院人体实验伦理委员会 3201000064763

2023 年 01 月 20 日
